# The role of the anterior intraparietal sulcus and the lateral occipital cortex in fingertip force scaling and weight perception during object lifting

**DOI:** 10.1152/jn.00771.2019

**Published:** 2020-07-15

**Authors:** Vonne van Polanen, Guy Rens, Marco Davare

**Affiliations:** ^1^Movement Control and Neuroplasticity Research Group, Department of Movement Sciences, Biomedical Sciences Group, KU Leuven, Leuven, Belgium; ^2^Leuven Brain Institute, KU Leuven, Leuven, Belgium; ^3^The Brain and Mind Institute, University of Western Ontario, London, Ontario, Canada; ^4^Department of Clinical Sciences, College of Health and Life Sciences, Brunel University London, Uxbridge, United Kingdom

**Keywords:** force scaling, parietal cortex, lateral occipital, TMS, weight perception

## Abstract

Skillful object lifting relies on scaling fingertip forces according to the object’s weight. When no visual cues about weight are available, force planning relies on previous lifting experience. Recently, we showed that previously lifted objects also affect weight estimation, as objects are perceived to be lighter when lifted after heavy objects compared with after light ones. Here, we investigated the underlying neural mechanisms mediating these effects. We asked participants to lift objects and estimate their weight. Simultaneously, we applied transcranial magnetic stimulation (TMS) during the dynamic loading or static holding phase. Two subject groups received TMS over either the anterior intraparietal sulcus (aIPS) or the lateral occipital area (LO), known to be important nodes in object grasping and perception. We hypothesized that TMS over aIPS and LO during object lifting would alter force scaling and weight perception. Contrary to our hypothesis, we did not find effects of aIPS or LO stimulation on force planning or weight estimation caused by previous lifting experience. However, we found that TMS over both areas increased grip forces, but only when applied during dynamic loading, and decreased weight estimation, but only when applied during static holding, suggesting time-specific effects. Interestingly, our results also indicate that TMS over LO, but not aIPS, affected load force scaling specifically for heavy objects, which further indicates that load and grip forces might be controlled differently. These findings provide new insights on the interactions between brain networks mediating action and perception during object manipulation.

**NEW & NOTEWORTHY** This article provides new insights into the neural mechanisms underlying object lifting and perception. Using transcranial magnetic stimulation during object lifting, we show that effects of previous experience on force scaling and weight perception are not mediated by the anterior intraparietal sulcus or the lateral occipital cortex (LO). In contrast, we highlight a unique role for LO in load force scaling, suggesting different brain processes for grip and load force scaling in object manipulation.

## INTRODUCTION

Every day we skillfully manipulate various objects with our hands. To lift an object skillfully, one has to precisely adjust fingertip forces to the weight of the object. That is, sufficient grip force, i.e., the force perpendicular to the object surface, has to be applied to avoid slipping of the object. In addition, the load force, i.e., the vertical force, has to overcome gravity and be equal to object weight in static object holding. Object weight can be predicted from visual object properties, such as size or material, to form an anticipatory motor plan and ensure a smooth lifting motion. Because feedback processes are slow, anticipatory scaling of fingertip forces results in more fluent lifts compared with feedback-driven movements. If the motor plan is incorrect, feedback processes during execution of the hand-object interaction, such as haptic perception of object movement or slip at the fingertips, can be used to quickly adapt the motor plan and apply the correct forces (Johansson and Westling 1988).

When the weight of an object cannot be inferred from object properties, one usually relies on previous lifting experience with that object, which is often referred to as sensorimotor memory (Johansson and Westling 1988). For instance, if a heavy object has been lifted, the next lift on an object with identical appearance will be scaled toward the heavy weight as well. The sensorimotor memory can be maintained for hours (Flanagan et al. 2001; Green et al. 2010; Nowak et al. 2007) and transferred between hands (Chang et al. 2008; Gordon et al. 1994; Nowak et al. 2005) and has a neural representation in the primary motor cortex (Chouinard et al. 2005; Loh et al. 2010).

The actual weight of an object can only be unequivocally determined after it has been lifted from the table. Recently, it has been suggested that sensorimotor memory effects could be linked to weight estimation. When an object was lifted after a heavy object, it was judged to be lighter than when lifted after a light one (van Polanen and Davare 2015b). Interestingly, force scaling parameters correlated with this perceptual bias, and both effects of force scaling and weight perception were stronger after longer sequences of lifts (i.e., increased magnitude of sensorimotor memory effects), suggesting that the corrections to fingertip forces and weight perception are associated. When forces are incorrectly planned based on the sensorimotor memory from a previous lift, these need to be updated appropriately. For instance, if a heavy object is expected although it is light, the planned forces will be too high and need to be downscaled to the actual weight. Possibly, these online corrections to the forces during object lifting bias the perception of object weight, leading, in this example, to a lower weight estimation. This perceptual bias depending on previous object weight has been replicated and was shown to transfer across hands (Maiello et al. 2018). Furthermore, a similar effect was shown for torque planning errors at object liftoff, which also affected heaviness and weight distribution estimation when lifting objects with asymmetric weight distribution (Schneider et al. 2019). All in all, these studies suggest a link between predictive lift planning and perception of object weight.

When acquiring sensory information about object weight, the loading and lifting phases of the movement might be especially important. These phases are dynamic because forces increase dynamically during the loading phase, and kinematics are dynamic after liftoff when the object is moved through the air. Since corrections to force planning based on sensory feedback mainly take place during the loading phase, i.e., between object contact and liftoff (Johansson and Flanagan 2009), it is possible that this phase is critical for building up a sensorimotor memory and the formation of a weight estimate. Indeed, it has been shown that a sensorimotor memory of force scaling is based on information acquired during the lifting phase, not the holding phase (van Polanen and Davare 2019). In addition, when observing lifting movements of others, the lifting phase was found to be most important for making judgments of object weight (Hamilton et al. 2007). Although these studies lack a common ground in the task used (lifting vs. observation), they both show that dynamic phases are important for weight perception. Finally, the relation between action planning and weight perception (Schneider et al. 2019; van Polanen and Davare 2015b) suggests that the loading and lifting phase could also be important for weight perception during the execution of object lifting. More specifically, van Polanen and Davare (2015b) showed that the perceptual bias was absent if objects were not lifted but a weight was passively pressed on the hand.

The neural pathways processing vision for action and perception have classically been divided into a dorsal and a ventral stream. More specifically, the dual stream theory proposes that visual information used for spatially locating the object and planning the action is processed in the dorsal stream, running from visual cortex to parietal areas, whereas information for object recognition is managed by the ventral stream, running from visual cortex toward the temporal cortex (Goodale and Milner 1992; Milner and Goodale 2008; Ungerleider and Mishkin 1982). A similar division between action-perception processing has been suggested for somatosensory perception (Dijkerman and de Haan 2007). It has also been argued that these visual streams do interact heavily (Cloutman 2013; Schenk and McIntosh 2010), especially as motor skill demands increase (van Polanen and Davare 2015a).

In the present study, we wanted to investigate the interplay between the dorsal and ventral streams during the execution of an action-perception task. We were particularly interested in how these streams mediate the effects of corrections to erroneously planned lifting actions and the biased weight perception. We focused on two key areas in the dorsal and ventral streams. First, we hypothesized that the anterior intraparietal sulcus (aIPS), part of the dorsal stream, could be an important node for controlling forces during object lifting and the correction of erroneously scaled forces. Indeed, this area is known to be involved in grip force scaling (Dafotakis et al. 2008; Davare et al. 2007). Regarding object perception, a similar status might be allocated to the lateral occipital cortex (LO), which is part of the ventral stream. We hypothesized that LO could mediate weight estimation, since this area is important for object recognition in both the visual and haptic modalities (Amedi et al. 2001, 2002) and is involved in the representation of object weight (Gallivan et al. 2014).

Furthermore, it is known that the temporal and parietal cortices are connected, based on primate (Borra et al. 2008, 2010; Distler et al. 1993) and human (Budisavljevic et al. 2018; Ramayya et al. 2010) studies, which could provide the basis for communication between dorsal and ventral streams. In addition, aIPS has been suggested to play an intermediate role in somatosensory action-perception interactions (Sedda and Scarpina 2012). Therefore, we hypothesized that aIPS and LO could be important nodes in the link between action and perception processes.

Previous studies have investigated the roles of aIPS and LO in grasp-to-lift movements by using transcranial magnetic stimulation (TMS). For instance, Cohen et al. (2009) showed that applying TMS at reach onset over aIPS altered reaching movement kinematics. TMS over LO also altered these movement kinematics, but only when grasping had to be performed based on memory of the object because grasping initiation was delayed, not with immediate grasping after object presentation. In addition, Dafotakis et al. (2008) showed that TMS over aIPS during reaching affected the corrective mechanisms when grip forces were incorrectly scaled based on sensorimotor memory. However, these studies stimulated before the actual hand-object interaction took place. As such, the online corrections during force scaling could not be investigated. Furthermore, it is unknown how these areas are involved in the perception of object weight when lifting a sequence of differently weighted objects.

The aim of the present study was to investigate the role of aIPS and LO in force scaling and weight perception. Specifically, we wanted to study how these areas are involved in how the relation between action corrections and perception is driven by previous experience, where force scaling correlated with weight estimation (van Polanen and Davare 2015b). To do this, participants performed an object lifting task and were asked to estimate object weight. To alter their force planning, we varied the order in which light and heavy objects were lifted. We were interested in how corrections would be applied when predictive force scaling was incorrect and how these incorrect predictions would affect weight perception. Moreover, TMS was applied to interfere with these processes. Importantly, we did not intend to use TMS to interfere with the formation of the sensorimotor memory of a previous lift but to interfere with corrective behavior when predictive force scaling based on the sensorimotor memory would be incorrect. Moreover, we expected that corrections to force scaling would lead to a perceptual bias and that TMS might interfere with this bias as well. TMS was given during the dynamic loading phase or the static holding phase, which was mainly added as control condition, to disrupt aIPS and LO and infer their causal role. We expected that disruption of aIPS might not only affect fingertip force scaling (Dafotakis et al. 2008; Davare et al. 2007) but also affect weight perception through connections with LO: When corrections need to be applied to the planned fingertip forces, aIPS might send information to LO, which could lead to perceptual weight biases. In return, LO might provide information to aIPS to scale fingertip forces based on known object weight information. More specifically, we expected that aIPS stimulation would reduce both the force corrections and the perceptual bias from previous lifting experience and alter the relation between force scaling and weight perception whereas LO would only affect the perceptual bias. Furthermore, we hypothesized that the involvement of these areas would be more pronounced in the dynamic loading phase compared with the static holding phase.

## METHODS

### Participants

Thirty right-handed participants took part in the study. They were divided into two groups, an aIPS stimulation group (8 men, 7 women; 21 ± 2.2 yr) and an LO stimulation group (6 men, 9 women; 21 ± 2.8 yr). Their right-handedness was accessed with the Edinburgh handedness questionnaire (Oldfield 1971), which provided a mean laterality quotient (LQ) of 90 ± 13. All participants gave written informed consent before participation, and the study was approved by the local ethical committee of the Biomedical Sciences Group at KU Leuven.

### TMS Procedure

**Fig. 1.**
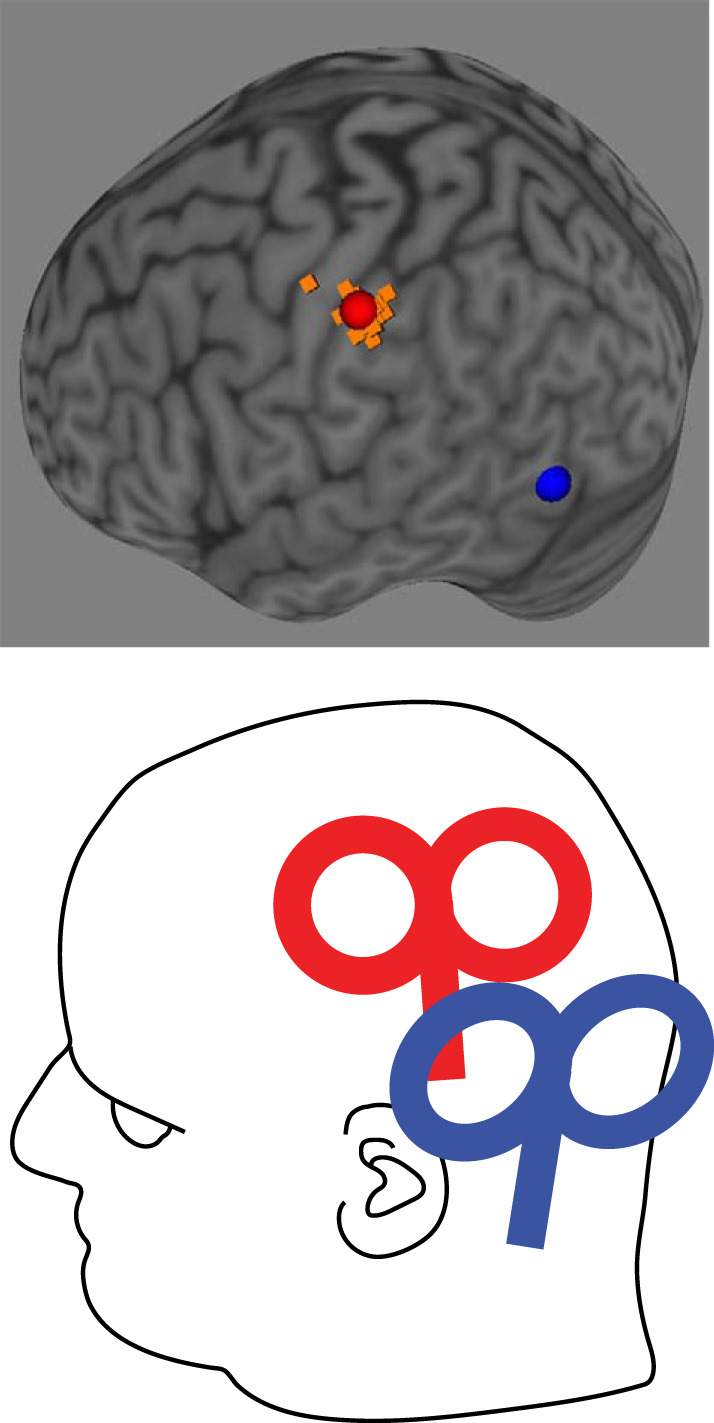
*Top*: brain areas targeted by transcranial magnetic stimulation (TMS). Spheres show the mean location on a standard Montreal Neurological Institute (MNI) brain: red, anterior intraparietal sulcus (aIPS); blue, lateral occipital cortex (LO). Orange squares represent individual stimulation locations for aIPS. Note that for LO coordinates from literature were used, which were the same for all participants. *Bottom*: example of TMS coil positioning for aIPS (red) and LO (blue).

The experiment was divided into two sessions. In the first session, participants were scanned in a 3-T MR scanner (Philips Achieva; Philips Healthcare). A high-resolution three-dimensional (3D) T1-weighted image was obtained with the following parameters: TR = 9.7 ms, TE = 4.6 ms, field of view = 256 × 256 mm^2^, 192 slices, voxel size = 0.98 × 0.98 × 1.2 mm^3^. The images were transferred to Brainsight (Rogue Research), which was used for online neuronavigation throughout the experiment. For the aIPS group, TMS stimulation sites were anatomically determined on the left hemisphere as the intersection between the postcentral and the intraparietal sulcus [see Frey et al. 2005; mean Montreal Neurological Institute (MNI) coordinates −46 ± 3, −37 ± 5, 46 ± 4]. The orientation of the TMS coil was positioned perpendicular to the intraparietal sulcus, with the handle pointing backward (Fig. 1). For the LO group, Talairach coordinates from literature (Amedi et al. 2001) were used, converted to MNI (−47, −61, −16), and located on the left hemisphere of individual structural MR images. For this group, a orientation similar to that in the aIPS group was used, with a slight clockwise rotation, if necessary, to avoid contact of the handle with the shoulder (see Fig. 1 for an example).

TMS was delivered with a 70-mm figure-of-eight TMS DuoMag XT coil (Deymed Diagnostic). Electromyography (EMG) signals of the first dorsal interosseus (FDI) muscle were recorded with adhesive electrodes in a belly-tendon montage. The motor evoked potentials (MEPs) in response to TMS over the left primary motor cortex (M1) were measured with the Brainsight system. In Brainsight, we defined the hotspot as the location on M1 that elicited the largest MEPs in the right-hand FDI muscle. The active motor threshold (aMT) was defined as the stimulation intensity when MEPs could be distinguished compared to background EMG in at least 5/10 stimulations while participants contracted the FDI muscle at submaximal levels. In the experiment, a stimulation level of 120% aMT was used. Average stimulation intensities were 48 ± 7% and 53 ± 9% of maximum stimulator output in the aIPS and LO groups, respectively. We used the aMT and not the rest motor threshold (rMT) because participants would be stimulated in the experiment during active movements and not rest. MEPs are higher during muscle contraction (Buccolieri et al. 2004), resulting in aMTs usually being lower than rMTs (Buccolieri et al. 2004; Ngomo et al. 2012). Hence, using the aMT value would be more tuned to the active nature of the task and minimize the risk of spread toward other areas (e.g., M1 in the case of aIPS stimulation).

### Task Procedure

**Fig. 2.**
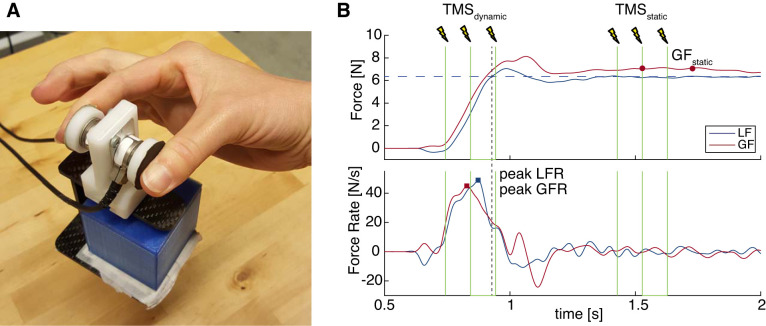
*A*: manipulandum with force sensors. Different objects can be placed in the basket to change object weight. *B*: example of trial with force traces of grip (GF) and load (LF) force (*top*) and force rates (GFR and LFR; *bottom*). Transcranial magnetic stimulation (TMS) was applied as a burst of 3 pulses at 10 Hz at contact (TMS_dynamic_) or 500 ms after liftoff (TMS_static_). Force parameters were calculated as peak force rates (peak LFR, peak GFR) and grip force during static holding (GF_static_; average between 600 and 800 ms after liftoff, red circles). Dashed vertical line indicates liftoff; dashed horizontal line indicates object weight.

Two force sensors (Nano17; ATI Industrial Automation) were used to record lifting performance of the participants. Force data was sampled in three directions at a 1,000-Hz frequency with a NI-USB 6343X (National Instruments). The sensors were attached to a manipulandum (Fig. 2*A*) that included a basket in which 3D printed cubes of different weights could be placed. The cubes were all of the same size (5 × 5 × 5 cm) but had different weights from being filled with different amounts of lead beads. A light (105 g) and a heavy (525 g) object were predominantly used in the experiment. To provide some variation in the weights, a dummy object of 317 g was presented in 10% of the trials. Finally, practice trials were performed with another object of 260 g. When the weight of the manipulandum (±120 g) was added, total weights of 2.2, 4.3, 6.3, and 3.8 N were obtained for the light, medium, heavy, and practice objects, respectively.

The participants were seated in front of a table. The manipulandum with the object was placed behind a switchable screen (Magic Glass) that could be in an opaque or a transparent state. In this way, the objects could be changed between trials by the experimenter unseen by the participant. Participants were instructed to grasp and lift the object with their right hand when the screen turned transparent and to hold it at a height of ~5 cm until the screen turned opaque again (±3 s) and then replace it back on the table (i.e., 1 “trial”). Participants were instructed to lift the object by placing the thumb and index finger on the force sensors. After they had replaced the object, they were asked to judge the weight of the object on a self-chosen scale with no constrained upper or lower limit.

During the participant’s movements, TMS was applied in two-thirds of the trials. Participants were instructed to ignore the TMS and continue their movement. TMS was always applied at 120% of aMT in a burst of 3 pulses at 10 Hz (total duration 200 ms). We used this burst of pulses to cover a larger time period of the loading phase. Similar protocols with bursts of 2–5 pulses at 10 Hz were used in previous studies (Davare et al. 2006; Rice et al. 2006; White et al. 2013). Three TMS conditions were used, TMS burst applied *1*) during dynamic loading or *2*) during static holding or *3*) no stimulation, which were presented in a pseudorandom order across the experiment. In the dynamic TMS condition (TMS_dynamic_) the first pulse was delivered when the participant contacted the object (i.e., grip force > 0.4 N). The three pulses together approximately covered the complete loading phase (see Fig. 2*B*). In the static TMS condition (TMS_static_), TMS was applied during static object holding, where the first pulse was delivered 500 ms after liftoff (load force > object weight). In the no-stimulation condition (TMS_no_), no TMS was applied during the trial. The TMS_no_ condition was included as a baseline condition to compare effects of TMS to unperturbed effects. In addition, the TMS_static_ condition served as control for timing-unrelated effects. That is, by providing TMS at two different time points we could control for side effects that were only due to the TMS itself. TMS triggering was controlled online based on sampled force data with custom-written programs in LabVIEW (National Instruments) and Signal software (Cambridge Electronic Design Limited). A trigger was sent to the TMS stimulator from a personal computer through the NI-USB 6343X, which was in turn connected to a micro1401–3 CED (Cambridge Electronic Design Limited).

Since we were interested in the effect of the previously lifted object on the present object, the order of object presentation was pseudorandomized for each participant. Each object order [light-light (LL), heavy-light (HL), light-heavy (LH), and heavy-heavy (HH)] was presented 10 times for each stimulation condition in a random order. Therefore, 120 trials were used for analysis. In addition, 14 dummy trials (10% total) with a medium weight were presented. These trials, the first trial, and the trials after dummy trials could not be analyzed because they had the wrong object order, which led to an extra 29 trials. For such trials, a stimulation condition was assigned randomly. In total, 149 trials were performed. Before the start of the experiment, the participant performed five practice trials to get familiar with the procedure, of which two trials included TMS (1 in the dynamic loading phase, 1 in the static holding phase).

### Data Analysis

Trials where TMS was not applied correctly (i.e., before lifting, not at all) or the object was not lifted were removed from the perceptual analysis (*n* = 16 trials, 0.44%). In addition, trials where objects were lifted multiple times or dropped or when force data collection failed were also removed from the force analysis (*n* = 25 trials, 0.69%).

Participants’ weight estimates were converted to *z* scores using the grand mean per participant and averaged for each object order, TMS condition, and TMS location.

Force data were filtered with a second-order low-pass Butterworth filter with a cutoff frequency of 15 Hz. Grip force (GF) was defined as the mean of the horizontal forces of both sensors, and load force (LF) was the sum of the vertical forces. GF onset and LF onset were defined as the time points at which the force crossed a threshold of 0.1 N. Note that the force threshold for triggering TMS_dynamic_ was slightly higher (0.4 N) to avoid responses to small initial bumps when grasping the object. Liftoff was the time point where LF overcame object weight. Grip force rate (GFR) and load force rate (LFR) were calculated as the first time derivatives of GF and LF, respectively. Since early force parameters are indicative of force planning, we were mostly interested in the peak force rates (Johansson and Westling 1988). The calculated force parameters are illustrated in Fig. 2*B*. Peak GFR and peak LFR were defined as the highest peak values of the force rates between GF onset and 50 ms after liftoff. Considering that we were primarily interested in corrections to force scaling and potential effects of TMS, we chose to focus on the maximum peak and not the first peak. Indeed, it has been shown that the first peak in the force rates is primarily indicative of force planning and does not account for corrective actions (Johansson and Westling 1988). Conversely, especially for the LH trials, corrective mechanisms might be explicitly visible in the maximum peak as the initial weight underestimation leads to a second larger peak. However, to explore predictive force planning, we also included the first peak values (peak1 GFR and peak1 LFR). To exclude small peaks due to noise, the first peak that was at least 30% of the maximum value was chosen. Finally, to investigate the time point at which the maximum force rate peak was reached, time to peak force rate parameters (time to peak GFR and time to peak LFR) were calculated as the time between GF onset and the peak force rate. Generally, longer time to peak values allow for more corrections to the force scaling.

Although these parameters are typical of most force scaling studies, they all occur at early phases in lifting (i.e., before liftoff), so they cannot be affected by TMS_static_, which occurs after this time point. Therefore, we also calculated a late force parameter, which was static grip force (GF_static_). Whereas during holding LF will normally not be much affected if the object does not move, the amount of GF can be more flexible while still maintaining a stable hold. We calculated GF_static_ as the average GF between 600 and 800 ms after liftoff, which was the period starting 100 ms after TMS_static_ was initiated (i.e., at the second pulse). This time period was chosen because it would be of the same duration as the stimulation period but was measured after the stimulation started so we could detect effects of stimulation on GF.

### Statistical Analysis

The variables of interest (perceptual answers, peak GFR, peak LFR, peak1 GFR, peak1 LFR, GF_static_, time to peak GFR, time to peak LFR) were analyzed with a mixed 2 (TMS location) × 3 (TMS condition) × 2 (previous weight) × 2 (current weight) analysis of variance (ANOVA). The factor TMS location was a between factor with the levels aIPS and LO for each participant group. The other factors were within factors, where TMS condition had three levels (TMS_dynamic_, TMS_static_, TMS_no_) and previous weight and current weight both had two levels (light or heavy). If a significant main effect or interaction with TMS location was found, the ANOVA was split into two 3 × 2 × 2 repeated-measures ANOVAs to further analyze effects in both TMS groups separately. If Mauchly’s test indicated a violation of the sphericity assumption, a Greenhouse–Geisser correction was applied. A Bonferroni correction was used for post hoc tests, which were performed with paired samples *t* tests or independent *t* tests for comparing within or between factors, respectively. The α-level was set at 0.05.

Although it was not the primary aim of our study, we also looked at effects of TMS in the previous trial on force scaling and perception in the current trial. Therefore, we performed a mixed 2 (TMS location) × 3 (TMS condition) × 2 (previous weight) × 2 (current weight) ANOVA on the perceptual answers, peak GFR, peak LFR, and GF_static_ for those trials. In this case, the TMS conditions (TMS_dynamic_, TMS_static_, TMS_no_) refer to simulation during the previous, not the current, trial. The TMS condition in the current trial was not taken into account for this analysis, as these effects were explored in our main analyses and we would have too few trials to analyze both effects simultaneously.

### Relation between Force Scaling and Perceptual Estimates

To investigate the relation between motor planning and weight perception, we performed linear mixed models to test the relation between force rates (peak LFR and peak GFR) and perceptual estimates. We performed three types of analyses, to investigate relations in effects of previous objects, TMS condition effects, and trial-by-trial variations, respectively.

First, to test whether motor and perceptual parameters were similarly affected in magnitude by previous objects, we compared sensorimotor memory effects (i.e., differences in force scaling with a previous light or heavy object) with perceptual biases (i.e., differences in weight perception with a previous light or heavy object). This analysis was performed to test whether differences between previous experiences in force scaling and perception would be related. A significant effect would indicate that participants with larger force differences would also have larger perceptual biases and vice versa. Since differences are in opposite directions for force scaling (higher force rates after heavy objects compared with light) and weight perception (lower estimates after heavy objects compared with light), negative predicted estimates in the model would be expected. We converted all variables (peak LFR, peak GFR, and perceptual estimates) to *z* scores. To calculate the difference according to the previous trial, we subtracted values with a previous light object from values with a previous heavy object. This was done for each TMS location and each TMS condition and for current light and heavy objects separately. If this obtained difference were different from zero, this would mean there was a difference in response to previous object weight. Next, to test whether these differences were related for the force parameters and weight perception, we used a mixed linear model to perform the regression analysis. The perceptual difference was the dependent variable, and peak LFR or peak GFR was entered as covariate. Current weight, TMS condition, TMS location, and peak LFR/GFR were used as fixed factors. Interactions between the factors and the intercept were also included in the model. A first-order autoregression was used for the covariate structure, and maximum likelihood estimation was used.

Second, we examined whether force and perceptual parameters were similarly affected in magnitude by TMS over a specific location. In contrast to the previous correlation, we did not compare differences according to previous object weight but used the difference between a TMS condition and the TMS_no_ condition. In this case, a significant effect would mean that participants with larger force differences in response to TMS would also show larger perceptual differences in response to TMS. For this analysis, we used the average *z*-scored variables (peak LFR, peak GFR, and perceptual estimates) for each participant and subtracted the baseline condition (TMS_no_) from the other TMS conditions (TMS_dynamic_ and TMS_static_) for each TMS location and current object weight separately. Because we did not find TMS effects on previous weight but only on current weight (see results), we did not calculate separate differences for previous weight conditions. We used the same mixed linear model for this analysis as for the previous weight differences.

Finally, we performed trial-by-trial comparisons. In this way, we could determine whether force scaling behavior was related to weight perception within individual participants on single trials. Here, we also performed a mixed linear model, similar to that described above. However, values for each trial were used instead of averages over trials.

## RESULTS

**Fig. 3.**
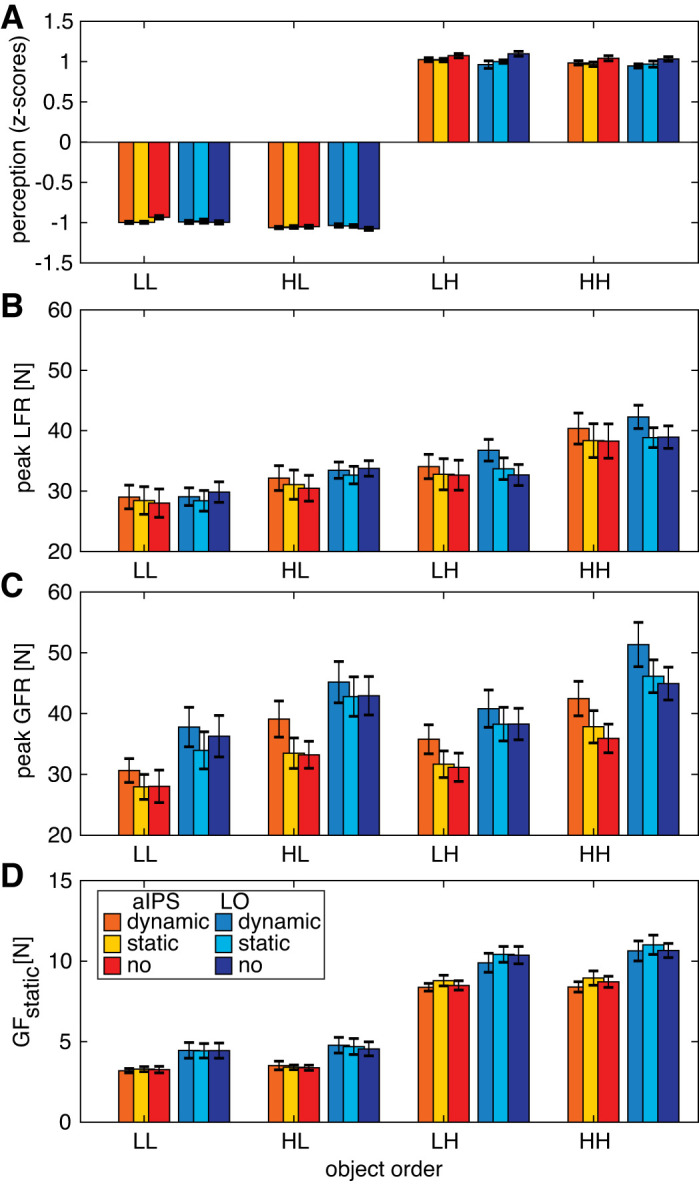
Results for perceptual estimates (*A*), peak load force rates (peak LFR; *B*), peak grip force rates (peak GFR; *C*), and grip force during static holding (GF_static_; *D*) for each object order [light-light (LL), heavy-light (HL), light-heavy (LH), heavy-heavy (HH)]. Colors indicate transcranial magnetic stimulation (TMS) group [red shades, anterior intraparietal sulcus (aIPS), blue shades: lateral occipital cortex (LO)] for the stimulation conditions TMS_dynamic_, TMS_static_, TMS_no_ (control condition). Error bars represent SE. Note that a main effect of previous weight was found for all parameters, which did not interact with TMS location or condition (mixed ANOVA). *N* = 15 participants per TMS location group.

**Fig. 4.**
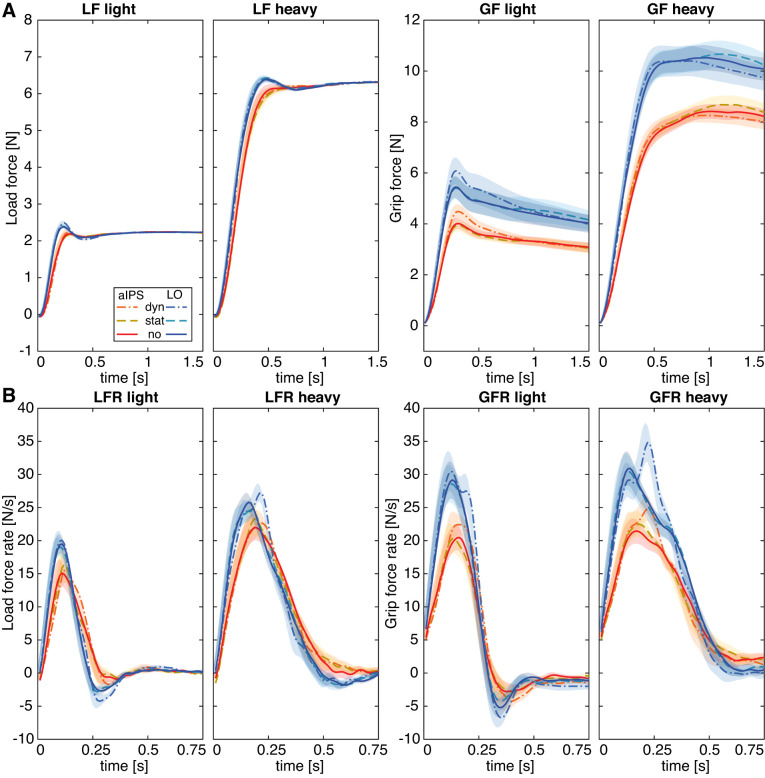
*A*: average force traces of load forces (LF) and grip forces (GF) for light and heavy objects separately. *B*: average traces of force rates (LFR and GFR) for light and heavy objects separately. Lines represent transcranial magnetic stimulation (TMS) conditions (dyn, TMS_dynamic_; stat, TMS_static_; no, TMS_no_) for the anterior intraparietal sulcus (aIPS) group (red shades) and the lateral occipital cortex (LO) group (blue shades). Shadings indicate SE. *N* = 15 participants per TMS location group.

**Fig. 5.**
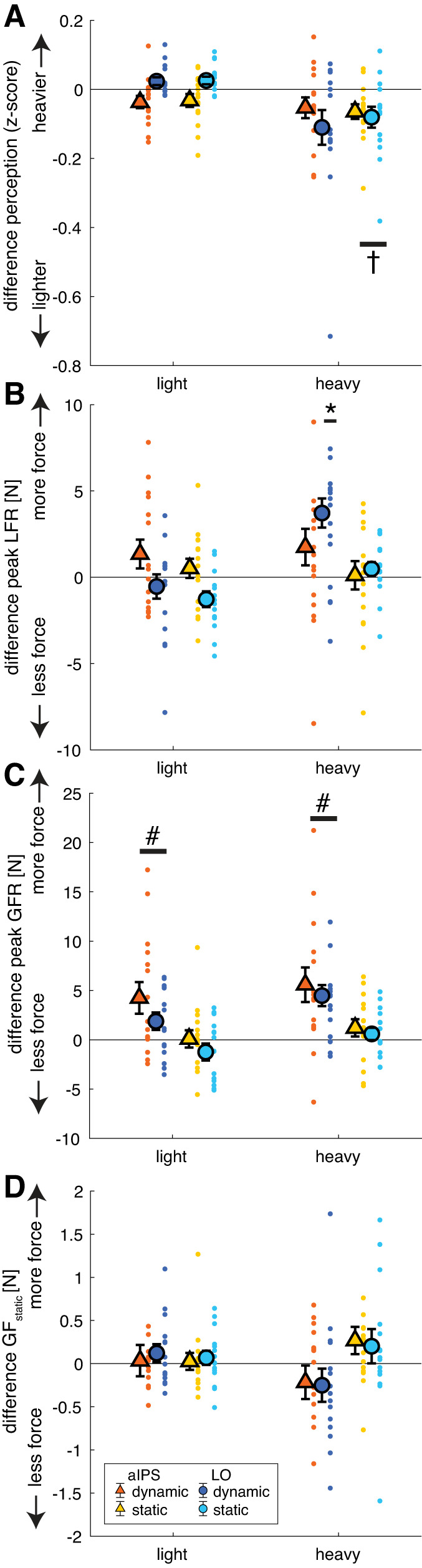
Differences between stimulation conditions (TMS_dynamic_ and TMS_static_) and the no-stimulation condition (TMS_no_) to display stimulation effects. *y*-Axes indicate whether objects are perceived to be heavier/lifted with more force after transcranial magnetic stimulation (TMS) compared with no TMS. Differences for perceptual estimates (*A*), peak load force rates (peak LFR; *B*), peak grip force rates (peak GFR; *C*), and grip force during static holding (GF_static_; *D*) are shown for light and heavy objects separately. Note that values are pooled over object order, since TMS did not interact with the effect of previous weight. Colors and symbols indicate TMS group [anterior intraparietal sulcus (aIPS), lateral occipital cortex (LO)] for the stimulation conditions (TMS_dynamic_ and TMS_static_). Error bars represent SE; dots indicate individual subjects. †Significant effect for TMS_static_, regardless of TMS location. *Significant effect for TMS_dynamic_ for LO only. #Significant effect for TMS_dynamic_, regardless of TMS location and object weight. *N* = 15 participants per TMS location group.

We investigated the role of aIPS and LO in force scaling and weight perception when lifting objects. TMS was applied in the dynamic loading phase (TMS_dynamic_), the static holding phase (TMS_static_), or not at all (TMS_no_, baseline condition). The results for the perceptual estimates and force parameters are shown in Fig. 3 and Table 1. Average force traces for each object and each TMS location and TMS condition are shown in Fig. 4. Differences with respect to the no-stimulation condition (TMS_no_) are illustrated in Fig. 5, to more clearly indicate the effects of stimulation. The ANOVA results for all the parameters can be found in Supplemental Table S1 (all Supplemental Material is available at https://doi.org/10.17605/OSF.IO/2G76U).

**Table 1.** Results for force parameters peak1 LFR, peak1 GFR, tpLFR, and tpGFR

	LL			HL			LH			HH		
	Dynamic	Static	No	Dynamic	Static	No	Dynamic	Static	No	Dynamic	Static	No
	*aIPS*											
Peak1 LFR	25.25 ± 2.38	25.04 ± 2.31	24.12 ± 2.55	28.85 ± 2.40	27.85 ± 2.86	26.52 ± 2.45	26.14 ± 1.89	26.82 ± 2.67	26.46 ± 2.97	32.73 ± 2.91	31.69 ± 2.80	31.36 ± 3.37
Peak1 GFR	25.74 ± 2.07	24.07 ± 2.48	24.21 ± 2.87	33.50 ± 2.85	29.55 ± 3.00	27.95 ± 2.54	29.52 ± 2.43	25.87 ± 2.67	25.53 ± 2.68	35.56 ± 2.95	32.42 ± 3.07	31.47 ± 2.86
tpLFR	0.17 ± 0.01	0.16 ± 0.01	0.16 ± 0.01	0.17 ± 0.01	0.17 ± 0.01	0.17 ± 0.01	0.24 ± 0.02	0.23 ± 0.02	0.23 ± 0.02	0.23 ± 0.01	0.23 ± 0.02	0.22 ± 0.02
tpGFR	0.18 ± 0.01	0.17 ± 0.01	0.18 ± 0.02	0.19 ± 0.01	0.18 ± 0.02	0.19 ± 0.01	0.25 ± 0.02	0.26 ± 0.03	0.25 ± 0.03	0.23 ± 0.02	0.24 ± 0.02	0.23 ± 0.02
	*LO*											
Peak1 LFR	26.30 ± 1.58	25.17 ± 2.04	26.76 ± 1.81	30.53 ± 1.80	29.59 ± 1.89	30.71 ± 1.84	28.36 ± 2.13	28.97 ± 1.91	27.72 ± 2.09	34.65 ± 2.15	34.38 ± 1.77	33.11 ± 2.01
Peak1 GFR	33.40 ± 3.45	31.30 ± 3.26	33.42 ± 3.68	40.97 ± 3.47	40.12 ± 3.66	39.31 ± 3.31	32.60 ± 3.31	33.74 ± 3.31	33.33 ± 3.12	44.10 ± 3.43	41.23 ± 2.83	41.16 ± 3.07
tpLFR	0.12 ± 0.01	0.12 ± 0.01	0.12 ± 0.01	0.12 ± 0.01	0.12 ± 0.01	0.12 ± 0.01	0.20 ± 0.01	0.18 ± 0.02	0.17 ± 0.01	0.19 ± 0.01	0.16 ± 0.01	0.17 ± 0.01
tpGFR	0.15 ± 0.01	0.13 ± 0.01	0.14 ± 0.01	0.15 ± 0.01	0.15 ± 0.01	0.15 ± 0.01	0.23 ± 0.02	0.22 ± 0.02	0.20 ± 0.02	0.21 ± 0.01	0.19 ± 0.01	0.19 ± 0.02

Values represent mean ± SE results for the force parameters first peak of load force rate (peak1 LFR), first peak of grip force rate (peak1 GFR), time to peak LFR (tpLFR), and time to peak GFR (tpGFR). for the anterior intraparietal sulcus (aIPS) and lateral occipital cortex (LO) groups. Columns are separated for object order (LL, light-light; HL, heavy-light; LH, light-heavy; HH, heavy-heavy) and transcranial magnetic stimulation (TMS) condition (TMS_dynamic_, TMS_static_, TMS_no_).

### TMS Affects Weight Perception but Not the Perceptual Bias

#### Behavioral effects.

The ANOVA on the perceptual weight estimates showed the expected main effect of current weight [*F*(1,28) = 66,786.5, *P* < 0.001, $\eta_{\text{p}}^{2}$ = 1.00] where light objects were rated lighter than heavy objects (Fig. 3). In addition, the effect of previous weight [*F*(1,28) = 11.7, *P* = 0.002, $\eta_{\text{p}}^{2}$ = 0.29] indicated that a perceptual bias was observed, where an object was perceived to be heavier when the previously lifted object was light (0.023 ± 0.01) compared with when it was heavy (−0.032 ± 0.01). This effect corroborates our previous findings, indicating a perceptual bias from previous lifted objects (van Polanen and Davare 2015b).

#### TMS effects.

The effect of previous weight did not interact with TMS condition or with TMS location, suggesting that the weight perception bias from the preceding object was not affected by TMS. However, we did observe a main effect of TMS condition [*F*(1.5,41.9) = 6.3, *P* = 0.008, $\eta_{\text{p}}^{2}$ = 0.18] and an interaction of current weight × TMS condition [*F*(1.7,46.5) = 6.1, *P* = 0.007, $\eta_{\text{p}}^{2}$ = 0.18]. Effects of stimulation are shown in Fig. 5*A* and show that, pooled for previous weight, weight estimation was lower in both stimulation conditions compared with TMS_no_. However, these results should be interpreted in light of the interaction of current weight × TMS condition. Post hoc tests of this interaction indicated that perceptual ratings were only lower after TMS_static_ compared with TMS_no_ and only for heavy objects (*P* = 0.004).

Since there was also a trend for a TMS location × TMS condition × current weight interaction [*F*(1.7,46.5) = 3.1, *P* = 0.065, $\eta_{\text{p}}^{2}$ = 0.10], we further explored this interaction by performing two separate repeated-measures ANOVAs on the TMS locations to shed light on these stimulation effects. For aIPS, we only found main effects [current weight: *F*(1,14) = 58,349.6, *P* < 0.001, $\eta_{\text{p}}^{2}$ = 1.00; previous weight: *F*(1,14) = 6.9, *P* = 0.020, $\eta_{\text{p}}^{2}$ = 0.33; TMS condition: *F*(2,28) = 5.0, *P* = 0.014,$\eta_{\text{p}}^{2}$ = 0.26]. Similar to the main effects of the mixed ANOVA, these effects indicated that objects were perceived as lighter when current objects were *1*) light compared with heavy, *2*) lifted after heavy objects compared with light objects, and *3*) after TMS_static_ compared with TMS_no_. No interaction effects were found in the aIPS group.

For the LO group, we found that the current weight × TMS condition effect was significant as well, in addition to the main effects of current weight and previous weight [current weight: *F*(1,14) = 23,310.3, *P* < 0.001, $\eta_{\text{p}}^{2}$ = 1.00; previous weight: *F*(1,14) = 4.8, *P* = 0.046, $\eta_{\text{p}}^{2}$ = 0.26; current weight × TMS condition: *F*(2,28) = 6.1, *P* = 0.007, $\eta_{\text{p}}^{2}$ = 0.30]. Further post hoc tests for this interaction revealed no significant effects of TMS condition after Bonferroni corrections. No significant differences were found between the two TMS groups on any TMS condition or current weight condition. Overall, although the perceptual bias induced by the previous lift was not affected by TMS, stimulation in the static phase, over either aIPS or LO, seemed to affect weight perception in the current lift.

### Early Force Parameters: LO Stimulation Affects Load Force Scaling

To test effects of TMS on parameters in the early phases of lifting (dynamic loading phase), we looked at the peak values of the force rates. Results for peak LFR are shown in Fig. 3*B*. The mixed ANOVA on peak LFR revealed main effects of current weight [*F*(1,28) = 171.8, *P* < 0.001, $\eta_{\text{p}}^{2}$ = 0.86], previous weight [*F*(1,28) = 208.5, *P* < 0.001, $\eta_{\text{p}}^{2}$ = 0.88], and TMS condition [*F*(1.3,35.2) = 9.8, *P* = 0.002, $\eta_{\text{p}}^{2}$ = 0.26]. In addition, interactions of current weight × previous weight [*F*(1,28) = 9.0, *P* = 0.006, $\eta_{\text{p}}^{2}$ = 0.24] and current weight × TMS condition [*F*(2,56) = 4.9, *P* = 0.010, $\eta_{\text{p}}^{2}$ = 0.15] and a triple interaction of current weight × TMS condition × TMS location [*F*(2,56) = 3.2, *P* = 0.048, $\eta_{\text{p}}^{2}$ = 0.10] were found.

#### Behavioral effects on peak LFR.

To start with the current weight × previous weight interaction, post hoc tests showed that all comparisons were significant (Fig. 3*B*). Peak LFR was lower for light objects compared with heavy objects (both previous weights: *P* < 0.001). In addition, objects that were lifted after a light object were lifted with a lower peak LFR compared with after heavy weights (both objects: *P* < 0.001). These results indicate that force scaling was based on the current weight and on previously lifted objects, for both light and heavy weights. Since all post hoc tests were significant, it is difficult to explain the interaction between current weight and previous weight. When looking at the values in Fig. 3, the interaction effect could be potentially explained by the notion that the effect of previous weight was somewhat stronger in heavy objects than light objects.

#### Effects of aIPS TMS.

To further investigate the triple interaction, we performed separate repeated-measures ANOVAs for the two TMS locations. No significant differences were found between the two TMS groups for any of the object weights or TMS conditions. In the separate ANOVA for the aIPS group, main effects of current weight [*F*(1,14) = 63.2, *P* < 0.001, $\eta_{\text{p}}^{2}$ = 0.82] and previous weight [*F*(1,14) = 83.9, *P* < 0.001, $\eta_{\text{p}}^{2}$ = 0.86] and an interaction of current weight × previous weight [*F*(1,14) = 6.2, *P* = 0.026, $\eta_{\text{p}}^{2}$ = 0.31] were found. These results were the same as those found in the mixed ANOVA (Fig. 3*B*), indicating that peak LFR was affected both by current and previous object. Stimulation effects, i.e., differences with respect to the TMS_no_ condition, are shown in Fig. 5*B*. The absence of effects of TMS condition suggests that TMS over aIPS did not influence peak LFR.

#### Effects of LO TMS.

For the LO group, we also found main effects of current weight [*F*(1,14) = 140.0, *P* < 0.001, $\eta_{\text{p}}^{2}$ = 0.91] and previous weight [*F*(1,14) = 129.1, *P* < 0.001, $\eta_{\text{p}}^{2}$ = 0.90]. However, for the LO group, we also found effects of TMS condition [*F*(1.2,16.7) = 10.3, *P* = 0.004, $\eta_{\text{p}}^{2}$ = 0.42] and a current weight × TMS condition interaction [*F*(2,28) = 9.6, *P* = 0.001, $\eta_{\text{p}}^{2}$ = 0.41]. Post hoc tests for this double interaction showed that in the TMS_dynamic_ condition a higher peak LFR was seen than in the TMS_static_ or TMS_no_ conditions, but only for heavy objects (both conditions, *P* < 0.014; see Fig. 5*B*). In other words, current heavy objects were lifted with higher load force rates when TMS was applied in the dynamic loading phase. The peak LFR differed between light and heavy objects for all TMS conditions (all *P* < 0.001).

#### Effects on time to peak LFR.

For time to peak LFR, main effects of current weight [*F*(1,28) = 246.7, *P* < 0.001, $\eta_{\text{p}}^{2}$ = 0.90], TMS condition [*F*(2,56) = 3.5, *P* = 0.037, $\eta_{\text{p}}^{2}$ = 0.11], and TMS location [*F*(1,28) = 8.7, *P* = 0.006, $\eta_{\text{p}}^{2}$ = 0.24] were found. In addition, there were interactions of current weight × TMS condition [*F*(1.6,46.7) = 4.9, *P* = 0.016, $\eta_{\text{p}}^{2}$ = 0.15] and current weight × previous weight [*F*(1,28) = 4.9, *P* = 0.035, $\eta_{\text{p}}^{2}$ = 0.15]. The main effect of location [*F*(1,28) = 8.7, *P* = 0.006, $\eta_{\text{p}}^{2}$ = 0.24] showed a longer time to peak LFR in the aIPS group compared with the LO group. Because of this main effect of location, we split the ANOVAs for the two TMS groups. For aIPS, only a main effect of current weight was found [*F*(1,14) = 115.0, *P* < 0.001, $\eta_{\text{p}}^{2}$ = 0.89], indicating that the time to peak LFR was longer for heavy objects compared with light ones (Table 1). For LO, main effects of current weight [*F*(1,14) = 136.6, *P* < 0.001, $\eta_{\text{p}}^{2}$ = 0.91] and TMS condition [*F*(2,28) = 4.1, *P* = 0.028, $\eta_{\text{p}}^{2}$ = 0.23] and an interaction of current weight × TMS condition [*F*(2,28) = 6.9, *P* = 0.004, $\eta_{\text{p}}^{2}$ = 0.33] were found. Similar to the effect of current weight in the aIPS group, post hoc tests indicated that peak LFR occurred later when lifting heavy objects than light objects in all stimulation conditions (all *P* < 0.001). Interestingly, similar to our findings for peak LFR, there was also an effect of TMS condition, where the time to peak LFR was longer after TMS_dynamic_ compared with TMS_no_ when lifting heavy objects (*P* = 0.009). This later LFR peak after stimulation in the dynamic loading phase is also visible in Fig. 4*B* (LFR heavy).

#### Effects on peak1 LFR.

The results for the first peak of LFR are shown in Table 1. The ANOVA on the first peak of the load force rates showed effects of current weight [*F*(1,28) = 53.9, *P* < 0.001, $\eta_{\text{p}}^{2}$ = 0.66] and previous weight [*F*(1,28) = 137.1, *P* < 0.001, $\eta_{\text{p}}^{2}$ = 0.83] and an interaction between current weight × previous weight [*F*(1,28) = 4.8, *P* = 0.036, $\eta_{\text{p}}^{2}$ = 0.15]. Similar to peak LFR, heavy objects were lifted with a higher peak1 LFR compared with light (both previous weights: *P* < 0.001) and a previous heavy object resulted in a higher peak1 LFR compared with a previous light lift (both current weights: *P* < 0.001). However, for peak1 LFR, no effects of TMS condition nor location were seen.

To summarize, it appears that TMS over aIPS did not influence load force scaling. On the other hand, LO stimulation delivered during the dynamic loading phase increased peak force rates, and their time to peak, only for heavy objects. When one looks at the force profiles in Fig. 4, it can be seen that after LO stimulation an extra peak is visible in the LF rates (Fig. 4*B*, LFR heavy), which could account for the increased maximum value at a later time point. Consequently, no effect of TMS was observed for the first LFR peak but only for the highest peak.

### Early Force Parameters: Grip Force Rates Are Affected by TMS but Not Specifically for TMS Location

#### Behavioral effects on peak GFR.

As expected, peak GFR was higher after lifting a previous heavy weight compared with a previous light weight [effect previous weight: *F*(1,28) = 204.9, *P* < 0.001, $\eta_{\text{p}}^{2}$ = 0.88; Fig. 3*C*]. In addition, effects of current weight [*F*(1,28) = 44.6, *P* < 0.001, $\eta_{\text{p}}^{2}$ = 0.61] and TMS condition [*F*(1.5,41.1) = 18.4, *P* < 0.001, $\eta_{\text{p}}^{2}$ = 0.40] and an interaction of current weight × TMS condition [*F*(2,56) = 3.6, *P* = 0.035, $\eta_{\text{p}}^{2}$ = 0.11] were found. Heavy objects were lifted with higher grip force rates than light objects in all TMS conditions (all *P* < 0.024). In accordance with the results on peak LFR, this indicates that grip forces were scaled according to both current and previous object weights.

#### TMS effects on peak GFR.

Furthermore, both for light and heavy objects, a higher peak GFR was seen after TMS_dynamic_ compared with TMS_static_ and TMS_no_ (all *P* < 0.023; Fig. 5*C*). The interaction effect of current weight × TMS condition could be explained by somewhat larger stimulation effects for heavy than light objects. A main effect of location was also found [*F*(1,28) = 4.4, *P* = 0.046, $\eta_{\text{p}}^{2}$ = 0.14], but separate ANOVAs showed the same main effects (all *F* > 8.0, all *P* < 0.003) for both TMS groups with no significant interaction effects. This indicated that there was just an overall difference in grip force rate that was higher in the LO group (Fig. 3*C*), independently of TMS condition. Considering this main effect of location (thus also including the TMS_no_ condition), these findings suggest that the LO group generated on average larger peak GFR values, indicating between-group differences rather than stimulation site differences. However, the peak GFR was increased when TMS was applied in the dynamic loading phase for both TMS groups.

#### Effects on time to peak GFR.

The results for the time to peak GFR are shown in Table 1. The mixed ANOVA on the time to peak GFR showed a main effect of current weight [*F*(1,28) = 60.0, *P* < 0.001, $\eta_{\text{p}}^{2}$ = 0.68] and an interaction of current weight × previous weight [*F*(1,28) = 16.4, *P* < 0.001, $\eta_{\text{p}}^{2}$ = 0.37]. Post hoc tests indicated that heavy objects had a longer time to peak GFR than light objects (both previous weights: *P* < 0.001). However, for light objects a previous light weight resulted in an earlier peak GFR compared with a previous heavy lift (*P* = 0.007), whereas this was reversed for heavy objects (*P* = 0.013). No effects or interactions with TMS condition or TMS location were found, indicating that the time to peak GFR was not affected by TMS.

#### Effects on peak1 GFR.

The results for peak1 GFR (Table 1) indicated that it was lower when lifting light objects compared with heavy ones [current weight: *F*(1,28) = 13.4, *P* = 0.001, $\eta_{\text{p}}^{2}$ = 0.32]. Furthermore, there were main effects of previous weight [*F*(1,28) = 170.7, *P* < 0.001, $\eta_{\text{p}}^{2}$ = 0.86] and TMS location [*F*(1,28) = 4.2, *P* = 0.050, $\eta_{\text{p}}^{2}$ = 0.13] and an interaction between those factors [*F*(1,28) = 4.4, *P* = 0.045, $\eta_{\text{p}}^{2}$ = 0.14]. Finally, a main effect of TMS condition was found [*F*(2,56) = 6.5, *P* = 0.003, $\eta_{\text{p}}^{2}$ = 0.19], where peak1 GFR was higher in TMS_dynamic_ compared with both TMS_no_ (*P* = 0.022) and TMS_static_ (*P* = 0.029).

Because of the main effect of TMS location and the interaction with previous weight, separate ANOVAs were performed for each TMS group. The effect of previous weight was present for both TMS locations [aIPS: *F*(1,14) = 43.3, *P* < 0.001, $\eta_{\text{p}}^{2}$ = 0.76, LO: *F*(1,14) = 187.9, *P* < 0.001, $\eta_{\text{p}}^{2}$ = 0.93]: The peak1 GFR was higher after lifting a heavy object than after a light object. When the TMS groups were compared for the TMS location × previous weight interaction, it was found that the LO group had higher first peaks than the aIPS group, but only when the previous lift was heavy (*P* = 0.031). Besides the effect of previous weight, no other significant effects were found for the LO group. On the other hand, in the aIPS group a significant effect of current weight was found [*F*(1,14) = 19.2, *P* = 0.001, $\eta_{\text{p}}^{2}$ = 0.58]. Finally, the main effect of TMS condition was also only seen in the aIPS group [*F*(2,28) = 5.0, *P* = 0.014, $\eta_{\text{p}}^{2}$ = 0.26]. However, none of the post hoc tests for the effect of TMS condition was significant in the aIPS group.

All in all, it seems that TMS in the dynamic loading phase affected the magnitude of the peak GFR but not the latency between force onset and peak GFR. Also, the effect of previous weight on GFR was not altered by TMS. Similar effects were seen for the first peak of GFR, although the effects of stimulation seemed weaker, as these effects were not significant in post hoc tests for the separate TMS locations. Since both LO and aIPS stimulation had the same effect on peak GFR, this suggests a nonspecific TMS effect rather than an actual indication of LO or aIPS contribution to grip force scaling. Because of these results, we also looked at TMS effects on grip force in the later phases of lifting to see whether these would also be similarly affected by TMS over both locations.

### Late Force Parameter: No Effects of TMS on Static Grip Force

Since the other force parameters all occurred before TMS_static_, they could not be affected by this stimulation during the static phase. Therefore, we also investigated GF_static_, which was the grip force during static holding. Main effects of current weight [*F*(1,28) = 1,276.0, *P* < 0.001, $\eta_{\text{p}}^{2}$ = 0.98], previous weight [*F*(1,28) = 12.7, *P* = 0.001, $\eta_{\text{p}}^{2}$ = 0.31], and TMS location [*F*(1,28) = 8.8, *P* = 0.006, $\eta_{\text{p}}^{2}$ = 0.24] were found. In addition, interaction effects of current weight × TMS condition [*F*(2,56) = 7.5, *P* = 0.001, $\eta_{\text{p}}^{2}$ = 0.21] and current weight × TMS location [*F*(1,0) = 4.5, *P* = 0.044, $\eta_{\text{p}}^{2}$ = 0.14] and a triple interaction of current weight × previous weight × TMS location [*F*(1,0) = 5.3, *P* = 0.029, $\eta_{\text{p}}^{2}$ = 0.16] were found.

#### Effect of aIPS TMS.

When the ANOVA was split for both TMS groups, only a main effect of current weight [*F*(1,14) = 757.4, *P* < 0.001, $\eta_{\text{p}}^{2}$ = 0.98] and an interaction of current weight × TMS condition [*F*(1.4,19.5) = 4.6, *P* = 0.034, $\eta_{\text{p}}^{2}$ = 0.25] were found for the aIPS group. This indicated that GF_static_ was higher for heavy objects than light ones (all conditions: *P* < 0.001, Fig. 3*D*), but post hoc tests did not show significant TMS condition effects. When observing Fig. 4*A*, *right*, the interaction might be explained by lower GF values after dynamic stimulation and higher values after static stimulation, for heavy objects only. However, these stimulation results were not statistically significant (Fig. 5*D*).

#### Effects of LO TMS.

For the LO group, main effects of current weight [*F*(1,14) = 570.6, *P* < 0.001, $\eta_{\text{p}}^{2}$ = 0.98] and previous weight [*F*(1,14) = 31.8, *P* < 0.001, $\eta_{\text{p}}^{2}$ = 0.70] and an interaction of current weight × previous weight [*F*(1,14) = 15.7, *P* = 0.001, $\eta_{\text{p}}^{2}$ = 0.53] were found. Post hoc tests revealed that GF_static_ was higher for heavy objects compared with light ones (both previous weights *P* < 0.001) and higher when a heavy object was previously lifted compared with a previous light lift (both current weights *P* < 0.008). There were no effects of TMS condition, indicating that LO stimulation did not influence GF_static_. Finally, further tests for the effect of TMS location revealed that the TMS groups differed significantly in the LH (*P* = 0.036) and HH (*P* = 0.012) conditions, again indicating that higher grip forces were on average used by the LO group. Since TMS location did not interact with TMS condition, this suggests a general group difference.

Since the effect of previous weight was only found in the LO group and not the aIPS group, this could suggest that aIPS stimulation eradicated the order effect on grip forces. However, since there was no interaction with TMS condition, indicating that this effect was also absent in TMS_no_ in the aIPS group, this result might merely reflect a general group difference without any stimulation effects.

### Limited Effects of TMS in the Previous Trial

As an exploratory analysis, we also looked at effects of TMS in the previous trial on force scaling and weight perception in the current trial. In this way, we could examine whether TMS interfered with the formation of a sensorimotor memory of a previous lift, which would alter predictive force scaling in the current trial. In addition, if haptic perception were disturbed in the previous trial this might not only alter the formation of the sensorimotor memory but could also lead to a different perceptual bias in the current trial. The results for this analysis are shown in Table 2. All ANOVA results are shown in Supplemental Table S2.

**Table 2.** Results for effects of TMS in the previous trial on parameters in the current trial

	LL			HL			LH			HH		
	Dynamic	Static	No	Dynamic	Static	No	Dynamic	Static	No	Dynamic	Static	No
	*aIPS*											
Perception	−0.98 ± 0.03	−0.96 ± 0.02	−0.97 ± 0.01	−1.05 ± 0.01	−1.05 ± 0.02	−1.06 ± 0.01	1.04 ± 0.02	1.04 ± 0.02	1.03 ± 0.03	1.02 ± 0.03	0.99 ± 0.03	0.99 ± 0.02
Peak LFR	28.11 ± 2.16	28.25 ± 2.22	29.32 ± 2.23	31.98 ± 2.54	31.46 ± 2.16	30.32 ± 2.00	34.00 ± 2.40	33.01 ± 2.41	32.57 ± 2.30	38.68 ± 2.45	38.63 ± 2.68	39.67 ± 3.01
Peak GFR	28.45 ± 2.25	27.63 ± 1.98	30.31 ± 2.04	34.89 ± 2.34	36.17 ± 2.67	34.84 ± 2.38	33.41 ± 2.04	32.31 ± 2.19	32.72 ± 2.40	38.04 ± 2.11	37.53 ± 2.41	40.75 ± 2.78
GF_static_	3.23 ± 0.15	3.14 ± 0.13	3.37 ± 0.16	3.40 ± 0.17	3.52 ± 0.18	3.40 ± 0.18	8.45 ± 0.23	8.42 ± 0.30	8.79 ± 0.31	8.56 ± 0.31	8.80 ± 0.45	8.73 ± 0.33
	*LO*											
Perception	−0.99 ± 0.02	−0.98 ± 0.02	−1.01 ± 0.02	−1.06 ± 0.02	−1.03 ± 0.02	−1.07 ± 0.02	1.06 ± 0.04	1.02 ± 0.03	0.97 ± 0.05	1.00 ± 0.02	0.97 ± 0.02	0.98 ± 0.04
Peak LFR	28.63 ± 1.43	29.14 ± 1.65	29.53 ± 1.84	33.21 ± 1.32	33.38 ± 1.46	33.26 ± 1.34	33.51 ± 1.60	33.97 ± 1.87	35.44 ± 1.82	39.85 ± 1.48	40.47 ± 1.83	39.29 ± 1.95
Peak GFR	36.37 ± 2.98	34.98 ± 3.13	37.20 ± 3.65	44.74 ± 3.40	43.16 ± 3.12	42.77 ± 3.45	38.77 ± 3.09	37.65 ± 2.44	40.43 ± 2.84	46.75 ± 2.75	49.18 ± 3.38	46.44 ± 2.79
GF_static_	4.42 ± 0.44	4.35 ± 0.44	4.62 ± 0.53	4.59 ± 0.44	4.79 ± 0.56	4.69 ± 0.46	10.03 ± 0.47	10.37 ± 0.54	10.31 ± 0.61	10.71 ± 0.55	10.83 ± 0.58	10.76 ± 0.51

Values represent mean ± SE results for the effects of transcranial magnetic stimulation (TMS) in the previous trial on parameters in the current trial. Data are shown for the perceptual estimations, peak load force rate (peak LFR), peak grip force rate (peak GFR), and grip force during static holding (GF_static_), for the anterior intraparietal sulcus (aIPS) and lateral occipital cortex (LO) groups. Columns are separated for object order (LL, light-light; HL, heavy-light; LH, light-heavy; HH, heavy-heavy) and TMS condition (TMS_dynamic_, TMS_static_, TMS_no_).

#### Weight perception is not affected by TMS in the previous trial.

Effects of current weight and previous weight were found [all *F*(1,28) > 10.4, *P* < 0.003, $\eta_{\text{p}}^{2}$ > 0.27]. Similar to the primary analysis, this showed that heavy objects were perceived to be heavier than light objects. In addition, when the previous lifted object was heavy, objects were judged to be lighter than when the previous object was light. These effects were not influenced by TMS.

#### Load force rates are not affected by TMS in the previous trial.

For peak LFR, main effects of current weight and previous weight were found as well as an interaction between these factors [*F*(1,28) > 9.3, *P* < 0.005, $\eta_{\text{p}}^{2}$ > 0.25]. All post hoc tests for the interaction were significant (all *P* < 0.001), indicating that peak LFR was higher after lifting heavy objects compared with light objects and when previously lifting heavy objects compared with light objects. Again, no effects of TMS were found on the load force rates.

#### Grip force rates are minimally influenced by TMS in the previous trial.

Main effects of current weight, previous weight, and TMS location were found [all *F*(1,28) > 4.4, *P* < 0.046, $\eta_{\text{p}}^{2}$ > 0.14]. In addition, an interaction between previous weight and TMS condition was observed [*F*(2,56) = 4.2, *P* = 0.020, $\eta_{\text{p}}^{2}$ = 0.13]. The main effects were similar to the primary analysis (see *Behavioral effects on peak GFR* and *TMS effects on peak GFR*), but the interaction between previous weight and TMS condition required further testing. First, similar to our previous findings, it was found that effects of previous weight were seen in all TMS conditions (all *P* < 0.001). However, in case of a previous object being light, peak GFR was higher when no stimulation was applied in the previous trial compared with TMS_static_. Second, further analyses from separate ANOVAs for the aIPS and LO groups were performed because of the main effect of TMS location. These revealed effects of current weight and previous weight for both locations [all *F*(1,28) > 11, *P* < 0.004, $\eta_{\text{p}}^{2}$ > 0.45]. An interaction of previous weight × TMS condition [*F*(2,28) = 6.0, *P* = 0.007, $\eta_{\text{p}}^{2}$ = 0.30] was found for the LO group, but no differences between the TMS conditions were found in the post hoc tests. For the aIPS group, a current weight × previous weight × TMS condition interaction [*F*(2,28) = 5.5, *P* = 0.010, $\eta_{\text{p}}^{2}$ = 0.28] was found. Post hoc tests revealed that the effect of TMS condition was only seen for the LL object order (*P* = 0.003). That is, when the aIPS group received TMS during the static phase of the previous lifting of a light object, they had lower peak GFR values during the current lifting of a light object compared with the no-stimulation condition. In addition, no effect of previous weight was found for light objects in the aIPS group in the TMS_no_ condition, whereas it was significant in the other conditions for this TMS group. Therefore, it appears that the difference might not have been caused by stimulation effects but more by the unexpected absence of a sensorimotor memory effect in the control condition.

#### Grip force in the static phase is not affected by TMS in the previous trial.

The analysis for GF_static_ showed main effects of current weight, previous weight, TMS condition, and location and an interaction between current weight × previous weight × TMS location [all *F*(1,28) > 3.7, *P* < 0.039, $\eta_{\text{p}}^{2}$ > 0.11). These effects indicated findings similar to the primary analysis (see *Late Force Parameter: No Effects of TMS on Static Grip Force*). However, the effect of TMS condition indicated that TMS_dynamic_ in the previous trial resulted in lower grip forces in the current trial compared with the TMS_no_ condition. However, the separate ANOVAs for each TMS location (which were performed because of the main effect of TMS location) showed no effects or interactions with TMS condition.

All in all, it seems that TMS applied on aIPS or LO during neither the dynamic loading phase nor the static holding phase induces behavioral changes in the next trial. That is, TMS did not interfere with the formation of a sensorimotor memory or the perceptual bias when applied during the previous trial. There might be a small effect in light objects for the planning of GFR, although the sensorimotor memory is not eradicated by TMS over aIPS or LO. It must be noted that TMS was also applied in the current lift, which could have interacted with any effects of TMS in the previous trial. However, we had too few trials to only investigate effects of TMS in the previous trial on current trials where no TMS was applied.

### Relations between Force Scaling and Perceptual Estimates

#### Effects of previous lifts are not related between force rates and perception.

We used linear mixed models to test relations between force scaling (peak LFR and peak GFR) and perceptual estimates. Since we were only interested in effects of peak LFR and peak GFR, only these results are reported. The full results for these analyses can be found in Supplemental Table S3.

As stated above, we found effects of previous object weight both for the force parameters and for the perceptual estimates. Therefore, we investigated whether these effects on the force parameters and the weight perception were associated. We calculated the differences between current trials with a previous light and a previous heavy lift. The linear mixed model showed that neither peak GFR differences nor peak LFR differences were significant predictors for the perceptual differences, nor were the other factors or interactions significant. Therefore, it seems that effects of previous object between force rates and perceptual biases were not related.

#### Stimulation effects of perception and peak LFR are related.

**Fig. 6.**
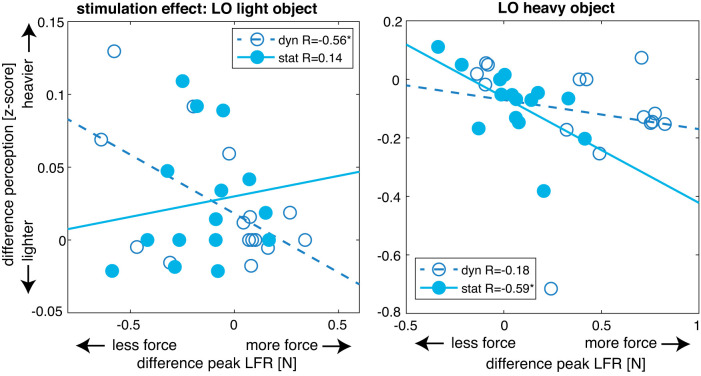
Correlations between stimulation effects (stimulation-TMS_no_) for peak load force rate (LFR) and perception. Significant correlations were found for TMS_dynamic_ (dyn) for a light object (*left*) but not TMS_static_ (stat), which is shown for comparison. Significant correlations were found for TMS_static_ for a heavy object (*right*) but not TMS_dynamic_, which is shown for comparison. Symbols indicate individual participants (*N* = 15 participants per TMS location group). Keys also display *R* values. Axes represent differences between previous heavy and previous light object conditions. *y*-Axes indicate whether objects are perceived to be heavier or lighter after lifting a heavy object. *x*-Axes indicate whether objects were lifted with more or less force after lifting a heavy object. *P < 0.05. LO, lateral occipital cortex.

To test whether force differences in response to TMS were related to differences in response to TMS for perceptual estimates, we calculated differences between trials with TMS and without TMS (TMS_no_ condition). Next, differences with respect to the TMS_no_ condition were used in a linear mixed model. Results showed that the peak GFR difference was not a significant predictor for perceptual differences. For the analysis with peak LFR differences, no significant effect of peak LFR differences was found either, but the interaction of TMS location × peak LFR [*F*(1,111) = 5.03, *P* = 0.027] and the triple interaction of TMS location × current weight × peak LFR [*F*(1,110) = 7.3, *P* = 0.008] were significant. Therefore, we decided to also perform separate correlations between the peak LFR differences and the perceptual differences for each condition (Table 3). Only significant correlations were seen in the LO group (see Fig. 6), where peak LFR correlated with perception in the TMS_dynamic_ condition for light objects (*R* = −0.56, *P* = 0.029) and in the TMS_static_ condition for heavy objects (*R* = −0.59, *P* = −0.019). These negative correlations for the LO group indicate that increases in peak LFR due to TMS are associated with decreases in weight perception. However, since stimulation in the static phase cannot affect peak LFR because it occurs after the measurement of this parameter, this correlation is unexpected. Furthermore, effects of TMS_dynamic_ on LO were found for heavy objects, not light objects. It is possible that the parameters, but not the stimulation effects, are related. In other words, variations in peak LFR could be related to variations in perceptual estimates.

**Table 3.** Between-subject correlations of stimulation differences (compared with no stimulation) between peak load force rates and perceptual estimates

	aIPS				LO			
	Light		Heavy		Light		Heavy	
	Dynamic	Static	Dynamic	Static	Dynamic	Static	Dynamic	Static
Peak LFR	–0.50 (–0.81 to 0.02)	–0.18 (–0.64 to 0.36)	0.37 (–0.18 to 0.74)	0.13 (–0.41 to 0.61)	–0.56* (–0.83 to –0.07)	0.14 (–0.40 to 0.61)	–0.18 (–0.64 to 0.36)	–0.59* (–0.85 to –0.12)

Between-subject correlations [*R* values: mean (95% confidence intervals)] of stimulation differences (compared with no stimulation) between peak load force rates (peak LFR) and perceptual estimates. *z*-Scored variables were averaged over object order, to obtain values for light and heavy objects, and differences with respect to no stimulation [transcranial magnetic stimulation (TMS)_no_] were calculated for TMS_dynamic_ and TMS_static_ separately. aIPS, anterior intraparietal sulcus; LO, lateral occipital cortex.

* *P* < 0.05.

#### Relationships between force rates and perception on individual trials.

Finally, we also performed a mixed linear model analysis on trial-by-trial variations for each participant. Here, for the analysis with peak GFR, we found a significant effect of peak GFR [F(1,3254) = 11.7, *P* = 0.001]. In addition, we found significant effects of TMS condition and current weight and interactions of TMS condition × current weight and TMS location × TMS condition × current weight. Because of these effects, we also performed separate analyses for each condition where we used peak GFR as the only factor. A significant effect was found in the TMS_static_ condition for the aIPS group [*F*(1,229) = 5.5, *P* = 0.02] and in the TMS_no_ condition for the LO group [*F*(1,268) = 9.4, *P* = 0.002]. The predictor estimates were −0.033 and −0.029, respectively, indicating negative associations between the force parameter and perceptual estimates.

For the analysis with peak LFR, a significant effect of peak LFR was found [F(1,3631) = 7.0, *P* = 0.008]. In addition, significant effects of TMS condition and current weight, an interaction between TMS condition × current weight, and an interaction of TMS location × TMS condition × current weight were found. Because of these effects, we performed separate analyses for all conditions with only peak LFR as fixed factor. Here, we only found a significant effect in the TMS_dynamic_ condition in the LO group for current light objects [*F*(1,262) = 7.7, *P* = 0.006], with a predictor estimate of −0.035. This negative predictor estimate indicates that higher force rate peaks are associated with lower weight estimations.

Overall, correlations were mainly found for the LO group and between peak LFR and weight perception. However, the correlations between forces and perceptual estimates were primarily observed for the light objects, whereas the results showed stimulation effects on weight perception and peak LFR for heavy objects only. Therefore, it is not clear whether stimulation had similar effects on both parameters or whether the correlations reflect a general relation between the parameters.

## DISCUSSION

In a recent study, we showed that sensorimotor memory effects were related to weight estimations (van Polanen and Davare 2015b). That is, both force scaling and weight perception were affected by the previously lifted object weight. Moreover, these effects correlated both across participants and in within-participant trial-by-trial comparisons. In the present experiment, we wanted to investigate the neural underpinnings of these effects in object lifting and weight perception. We hypothesized a role for aIPS, known to be involved in force scaling (Dafotakis et al. 2008; Davare et al. 2007), and LO, which is important in object perception (Amedi et al. 2001), and used TMS to infer their causal role. Whereas we replicated the effects of object order for both force scaling and weight perception, we did not find strong associations between action and perception components. Furthermore, although we did find effects of both aIPS and LO stimulation on force scaling and weight perception, these stimulation effects did not alter the effect of previously lifted objects on force scaling and weight perception. Therefore, although these areas might play a role in object lifting and weight perception, they do not seem to mediate effects of force planning based on previous experience on lifting performance and weight estimation. In the next paragraphs, we further elaborate on these findings.

First of all, we did replicate effects of object order on force scaling and weight perception. When a heavy object was previously lifted, force scaling was larger than when the previously lifted object was light. This corroborates studies showing that forces are planned based on the sensorimotor memory of previous objects (Gordon et al. 1994; Johansson and Westling 1988; Loh et al. 2010; van Polanen and Davare 2015b). In addition, we showed that when a heavy object was previously lifted, the current object felt lighter than when a light object was previously lifted, replicating the perceptual bias found in previous studies (Maiello et al. 2018; van Polanen and Davare 2015b). In our previous study, we only found an effect for current light objects, not heavy objects (van Polanen and Davare 2015b). In that study, we argued that for heavy objects differences needed to be larger to be perceptually discriminable. The findings in this study indicate that the perceptual bias can be seen for both light and heavy objects since we found no interaction of previous weight with current weight. Since the present study has a larger power (*N* = 30 participants) compared with the previous study (*N* = 10 participants), this might explain why we could detect an effect for both object weights. However, although the biases were significant, they were not very large. On average, participants perceived the light object to be 7% (0–19%) lighter after lifting a heavy object compared with lifting the same light object. The heavy object was perceived as 5% (−14–21%) heavier when it was lifted after a light object compared with being lifted after the same heavy object. Differences in object weight that can be perceived have been found to range between 3% and 12% (Jones 1986). Although the perceptual biases are small on average, they would be detectable for participants. Because of the individual differences, the effect might have been clearer for some participants than others.

In contrast to our previous study, we only found few correlations between force scaling parameters and perception. For comparisons across participants, effects of previous object weight on force scaling parameters did not seem to be associated with such effects on perceptual estimates. For within-participant trial-by-trial comparisons, significant relations were found, but analyses for separate conditions revealed few significant effects. Although few studies have compared these order effects on action and perception components (Schneider et al. 2019; van Polanen and Davare 2015b), the lack of correlation in this study casts doubt on the hypothesis that these effects stem from a common underlying mechanism. It would suggest that the relation is weak or not very robust. More specifically, the TMS procedure could have weakened the relation between force control and perceptual effects. Further research is needed to provide more insight into the involved processes. In the present study we investigated the role of the aIPS and LO, but these areas do not seem to mediate effects of previous lifting experience on force lifting or weight perception during hand-object interactions, or on the potential association of these effects.

Several other areas could be proposed to play a role in force planning based on previous experience. It has been well established that the sensorimotor memory of previous lifts is represented in the primary motor cortex (M1) (Berner et al. 2007; Chouinard et al. 2005; Loh et al. 2010; Nowak et al. 2005). However, it is likely that M1 receives input from other areas (Parikh et al. 2014). For instance, it has been shown that effects on grip force scaling can be differently affected depending on the timing of M1 stimulation (Berner et al. 2007). Furthermore, it is known that inputs to M1 change based on grasp type, through connections with the ventral premotor area (PMv) and indirectly from aIPS (Davare et al. 2007, 2008). For these areas, it was shown that PMv plays a role in predictive force scaling according to recent lifts, whereas aIPS is involved in force adjustments (Dafotakis et al. 2008). Functional MRI studies have shown more areas that seem to be involved in unpredictable weight changes in fronto-parietal circuits (Schmitz et al. 2005) but also the primary somatosensory cortex (Jenmalm et al. 2006). Therefore, it looks like a network of areas is involved in force planning according to previous weight experience. Possibly, aIPS is more concerned with online corrections of movements, as has been observed in grasping tasks (Glover et al. 2005; Rice et al. 2006; Tunik et al. 2005) or force adjustments in lifting tasks (Dafotakis et al. 2008), but less with predictive scaling according to previous experience. Finally, it is possible that the stimulation during the dynamic loading phase was already too late to interfere with the retrieval of a sensorimotor memory. It is likely that the motor plan encoding fingertip forces is already formed based on the sensorimotor memory before contacting the object, and TMS during lifting would not cause changes in this motor plan. Indeed, no effects of TMS were found for the first peak of the load force rates. Although an effect of TMS was still seen for the first peak of the grip force rates, in post hoc tests these effects were not significant. In a previous study, TMS was applied in the reaching phase and it was found that aIPS was involved in force adjustments to incorrectly predicted object weights (Dafotakis et al. 2008). In the present study, we were interested in the corrections that would be applied in response to an incorrect force scaling from previous experience and its effect on weight perception. Therefore, we chose to stimulate during the loading phase, during which those corrections take place.

Although our primary aim was to examine effects of previous experience on force scaling, and thus on the retrieval, not the formation, of a sensorimotor memory, our data allowed for an analysis of TMS effects on the formation of a sensorimotor memory. These results did not show that TMS in the previous trial led to the alteration of sensorimotor memory effects. Hence, we found no evidence that aIPS or LO played a role in this process, suggesting that these areas do not play a role in either the acquisition or the retrieval of the sensorimotor memory during object lifting movements. It must be noted that stimulation in the current trial could have masked effects of stimulation in the previous trial. However, our data set had too few trials to investigate effects of previous stimulation on current trials without any TMS.

The neural network of weight perception is less clear from the literature. Some studies indicate that weight is represented in LO (Gallivan et al. 2014). Other studies suggest roles for M1 in representing weight (Chouinard et al. 2009) and sense of effort in force production (Takarada et al. 2014). However, it is not exactly clear whether these findings also hold for judging weight. The present findings indicate that although aIPS and LO did not seem to be involved in the perceptual bias from previous lifted objects, an effect on weight perception by TMS, independent of stimulation site, was observed. When TMS stimulation was applied during the static phase, heavy objects were judged to be lighter. Since there are connections between parietal and temporal areas (Budisavljevic et al. 2018; Ramayya et al. 2010), it is possible that perceptual processing of weight runs through connections between these areas. Interestingly, a trend was observed for a specific object weight effect for LO stimulation: if LO was stimulated, heavy objects appeared to be lighter, whereas light objects were not perceived differently. Such a specific weight effect was not seen after aIPS stimulation. This result could be interpreted as a decrease in weight discrimination, where the different objects could be less well discriminated in weight, i.e., making heavy objects appear lighter and light objects appear heavier. This would indicate that LO is involved in weight discrimination. Although this is a speculative conclusion from our study results, this is an interesting notion that could be further investigated in future studies where more different weights should be measured.

More remarkably, we did find a similar object weight-specific effect of LO stimulation on load force rates: when LO was stimulated in the dynamic loading phase, load force rates increased only for heavy objects. Such an effect was not seen in aIPS, where TMS over this area did not affect load force rates. This suggests that LO is specifically involved in the scaling of load forces. In line with the narrative on weight discrimination, it seems that load force scaling was also less discriminative for object weight after LO stimulation. Correlations between stimulation effects on weight estimation and load force rates suggest that these effects could indeed be related. However, few correlations were seen and might not reflect stimulation effects but general similarities between load forces and weight estimation. In addition, correlations between parameters were mainly found for light objects, whereas effects of TMS were mainly seen for heavy objects. Furthermore, when considering stimulation differences, no main effect of peak LFR was found for predicting perceptual estimates in the linear mixed model. Therefore, these correlations should be interpreted with care.

The effects on grip forces were not in line with effects of weight estimation or load force rates. Grip force rates increased after stimulation of either aIPS or LO, whereas static grip force was not significantly affected. Whereas previous research indicated that the amount of grip force applied during holding was related to weight estimation (Ellis and Lederman 1999; Flanagan and Bandomir 2000; Flanagan and Wing 1997), these might be governed by areas other than aIPS or LO as investigated in this study. Although stimulation to both these areas increased grip force rates and reduced weight estimation, this effect was different regarding object weight and stimulation timing. The perceptual effect was only seen in heavy objects after stimulation in the static phase, whereas grip force rates were higher after stimulation in the dynamic loading phase for both light and heavy objects. It could be argued that the effect on grip force rates for both areas indicates a nonspecific TMS effect rather than an actual indication of involvement of these areas (see discussion of study limitations below). However, the effect on grip forces we found was timing specific: only TMS applied in the dynamic loading phase increased grip forces, but not when applied in the static holding phase. In a previous study, a contribution of aIPS to grip force scaling was only found in a specific time window of 120–170 ms before object contact (Davare et al. 2007). The present results suggest that after object contact both aIPS and LO play a role in the online control when grip forces increase but not any longer when stable force levels are reached.

As mentioned above, whereas stimulation to both areas affected grip forces, load forces were only altered when TMS was applied over LO, not aIPS. This suggests that different processes are involved in online control of grip and load forces. In general, it has been assumed that grip and load forces are tightly coupled in an anticipatory manner (Flanagan and Wing 1993; Johansson and Westling 1984, 1988), suggesting they are controlled by the same neural network. Recently, however, it was shown that this coupling can be intermittent, not continuous (Grover et al. 2018). Although both force components are important for object lifting, there are slight differences in their functionality. Grip forces are needed to ensure stability of the grasp to avoid slipping, which means they have to be adjusted to both object weight and surface friction and can contain a variable safety margin while still maintaining a stable grip. Load forces only need to be adjusted to object weight, which suggests a tighter link with weight perception, as indicated by our results. It must be noted that previous studies have found involvement of several brain areas for grip forces, but not load forces, such as left supplementary motor area (White et al. 2013), left aIPS (Davare et al. 2007), and dorsal premotor area (van Nuenen et al. 2012). To our knowledge, the neural correlates specifically tuned to load force scaling are less clear. Here we show for the first time an influence of a perceptual processing area, i.e., LO, on the control of load forces. Possibly, load and grip forces are generated with input from different brain areas and coupled together in others. In the literature, different areas have been suggested to be involved in this coupling, such as the cerebellum (Kawato et al. 2003), M1 (Schabrun et al. 2008), and the right intraparietal cortex (Ehrsson et al. 2003).

Finally, we hypothesized that the dynamic loading phase of object lifting would be more influential on weight perception than the static phase. Regarding force scaling, it has been shown that the lifting phase is important in building up a sensorimotor memory (van Polanen and Davare 2019). Furthermore, weight perception is influenced by object size when the size is visually shown during lifting, but not holding, and this effect reappears when the object is replaced (Plaisier et al. 2019). However, we did not find stronger effects of TMS on weight perception when it was applied in the dynamic loading phase compared with the static holding phase. Instead, significant effects were only seen for stimulation in the static phase for heavy objects. It is remarkable that weight perception was affected long after it could have been perceived for the first time (i.e., 500 ms after liftoff). The study of Plaisier et al. (2019) does indicate that a weight estimate can be formed 300 ms after liftoff, but here we show that it can be affected even later. This could suggest that weight estimation is not formed at a specific time point but can be constructed over a longer period during hand-object interactions. Although this effect during static holding does not negate the importance of the dynamic loading phase for weight perception, it seems that LO and aIPS have a stronger influence on weight estimation in later phases of lifting.

The stimulation procedure used in this study may also have some limitations. Because stimulation was provided randomly across trials, it is possible that TMS in the previous trial affected the storage of information differently for action and perceptual processes, thereby decreasing the relation between the two processes even on the following no-stimulation trials. Our exploratory analysis of TMS in the previous trial suggests that TMS did not alter the formation of a sensorimotor memory and perceptual bias in the previous trial. However, we had too few trials to investigate the effects of stimulation in previous trials on the relation between force scaling and weight perception in trials without TMS during the current lift.

Furthermore, another limitation of the present study is that no sham stimulation condition was used. We used a control condition without stimulation to access normal baseline effects. Therefore, placebo effects of stimulation, such as auditory, somatosensory, or startling effects from the TMS pulse, cannot be ruled out. Since we found effects of both aIPS and LO stimulation on grip forces, this could be due to a stimulation side effect. More specifically, participants could have squeezed more in response to the TMS, thereby increasing their grip force rates. We used two different TMS timings to control for such TMS side effects. Indeed, we only found an effect on grip forces when applying TMS in the dynamic loading, not the static holding, phase. However, this does not completely rule out the possibility of a placebo effect in early phases of lifting because grip forces in dynamic and static phases might be differently susceptible to TMS. Further studies using appropriate sham conditions should rule out these possibilities.

Finally, since we only used two object weights in the study, the interpretation of differences between light and heavy objects is limited. Since the aim of the study was to investigate effects of previous lifts, using two weights was appropriate. However, to make definite conclusions about the effects of changes in weight discrimination and tuning of load forces to specific object weights, a larger range of object weights should be used.

To conclude, aIPS and LO do not seem to be involved in mediating force scaling and weight judgment according to previous object experience. Whereas both areas appear to play a role in grip force scaling, LO specifically contributes to load force scaling, possibly related to object weight discrimination. This suggests that grip force and load forces are processed differently and both force components might be differently related to weight perception. More research is needed to shed more light on the relation between the involvement of LO in load force scaling and weight perception and its potential role in weight discrimination.

## GRANTS

This research was supported by Fonds Wetenschappelijk Onderzoek grants (FWO postdoctoral fellowship, Belgium, 12X7118N; FWO Odysseus, Belgium, G/0C51/13N).

## DISCLOSURES

No conflicts of interest, financial or otherwise, are declared by the authors.

## AUTHOR CONTRIBUTIONS

V.v.P. and M.D. conceived and designed research; V.v.P. and G.R. performed experiments; V.v.P. analyzed data; V.v.P. and G.R. interpreted results of experiments; V.v.P. prepared figures; V.v.P. drafted manuscript; V.v.P., G.R., and M.D. edited and revised manuscript; V.v.P., G.R., and M.D. approved final version of manuscript.
